# A bispecific antibody targeting HER2 and PD-L1 inhibits tumor growth with superior efficacy

**DOI:** 10.1016/j.jbc.2021.101420

**Published:** 2021-11-16

**Authors:** Yi-Li Chen, Yue Cui, Xinyuan Liu, Guojian Liu, Xingchen Dong, Lei Tang, Yifeng Hung, Chunhe Wang, Mei-Qing Feng

**Affiliations:** 1 Department of Biological Medicines & Shanghai Engineering Research Center of Immunotherapeutics, Fudan University School of Pharmacy, Shanghai, China; 2 Department of Reasearch and Development Center, Dartsbio Pharmaceuticals Ltd, Zhongshan, Guangdong, China; 3 Department of Antibody Discovery, Shanghai Mabstone Biotechonology, Ltd, Shanghai, China; 4 Biotherapeutics Discovery Research Center, Shanghai Institute of Materia Medica, Chinese Academy of Sciences, Shanghai, China

**Keywords:** human epidermal growth factor receptor 2 (HER2), programmed death ligand 1, breast cancer, antibody-dependent cell-mediated cytotoxicity, ADCC, antibody-dependent cell-mediated cytotoxicity, BC, breast cancer, BLI, biolayer interferometry, BsAbs, bispecific antibodies, BTC, biliary tract cancer, CDC, complement-dependent cytotoxicity, CRC, colorectal cancer, DC, dendritic cell, DSF, differential scanning fluorimetry, GC, gastric cancer, GEJC, gastroesophageal junction cancer, HER2, human epidermal growth factor receptor 2, mAbs, monoclonal antibodies, MLR, mixed lymphocyte reaction, MOA, mechanisms of action, NSCLC, non-small-cell lung cancer, PBMC, peripheral blood mononuclear cell, PD-1/PD-L1, programmed cell death protein 1 and programmed cell death ligand 1, SDS-PAGE, sodium dodecyl sulfate–polyacrylamide gel electrophoresis, SEC, size exclusion chromatography, VH, variable heavy, VL, variable light

## Abstract

Activation of the programmed cell death protein 1 and programmed cell death ligand 1 (PD-1/PD-L1) signaling axis plays important roles in intrinsic or acquired resistance to human epidermal growth factor receptor 2 (HER2)-directed therapies in the clinic. Therefore, therapies simultaneously targeting both HER2 and PD-1/PD-L1 signaling pathways are of great significance. Here, aiming to direct the anti-PD-L1 responses toward HER2-expressing tumor cells, we constructed a humanized bispecific IgG1 subclass antibody targeting both HER2 and PD-L1 (HER2/PD-L1; BsAb), which displayed satisfactory purity, thermostability, and serum stability. We found that BsAb showed enhanced antibody-dependent cell-mediated cytotoxicity (ADCC) activity *in vitro*. In the late phase of peripheral blood mononuclear cell (PBMC)-humanized HER2^+^ tumor xenograft models, BsAb showed superior therapeutic efficacies as compared with monoclonal antibodies (mAbs) or combination treatment strategies. In cynomolgus monkeys, BsAb showed favorable pharmacokinetics and toxicity profiles when administered at a 10 mg/kg dosage. Thus, HER2/PD-L1 BsAb was demonstrated as a potentially effective option for managing HER2^+^ and trastuzumab-resistant tumors in the clinic. We propose that the enhanced antitumor activities of BsAb *in vivo* may be due to direct inhibition of HER2 signaling or activation of T cells.

Human epidermal growth factor receptor-2 (HER2, HER2/neu, or human ERBB-2) belongs to a family of four-transmembrane receptors involved in the regulation of cell growth and differentiation (1). HER2 is overexpressed in multiple solid tumors including breast (BC), colorectal (CRC), gastric (GC), gastroesophageal junction (GEJC), non-small-cell lung (NSCLC), biliary tract (BTC), and bladder cancers. The emergence of trastuzumab (Herceptin), a widely used monoclonal antibody (mAb) therapy that blocks HER2 signaling and induces antibody-dependent cell-mediated cytotoxicity (ADCC) toward HER2^+^ tumors, has significantly improved the survival rate of HER2^+^ carcinoma patients (2, 3, 4, 5). However, approximately 50% to 80% of HER2^+^ BC patients would benefit from trastuzumab, while the rest either show no response throughout the treatment or develop drug resistance posttreatment (6).

Known causes of trastuzumab-induced drug resistance include downregulation of HER2, upregulation of PD-L1 expression in tumor cells, or mutations in the HER2-PI3K-AKT signaling pathway (7, 8, 9). PD-L1 is highly expressed in various types of tumor cells, and its counter receptor PD-1 is typically expressed in various immune cells, such as tumor infiltrating T cells. Their interaction mediates different mechanisms of immune escape, including T cell apoptosis, functional inhibition, and exhaustion (10, 11, 12, 13). It has been previously reported that trastuzumab treatment can increase both PD-1 and PD-L1 expression levels in clinical and preclinical models (8, 14). It is thus believed that the upregulation of PD-L1 is, at least partially, the cause for induced drug resistance of HER2^+^ tumors to trastuzumab.

The clinical benefits of antagonistic mAbs against PD-1 or PD-L1 have been demonstrated in a variety of human cancers (15, 16, 17). However, a substantial number of patients fail to respond to anti-PD-1 or anti-PD-L1 monotherapies (18, 19). Preclinical and clinical data have demonstrated the effectiveness of trastuzumab and anti-PD-1 combination therapy in managing HER2^+^ cancers (20, 21, 22). In trastuzumab-refractory HER2^+^ metastatic BC patients, a combination of trastuzumab, pembrolizumab, and chemotherapy resulted in long-lasting clinical responses (20), demonstrating the power of targeting HER2 and PD-1/PD-L1 signaling pathways simultaneously.

Bispecific antibodies (BsAbs) that recognize two separate epitopes or antigens by design are a rapidly growing class of cancer therapeutics. The advantages of BsAbs over mAb and combination therapies include acting on two synergistically related or complementary signaling pathways, increasing affinity to tumor tissues expressing both targets, recruiting target one-expressing effector cells to target two-expressing tumor tissues, and/or reducing drug resistance caused by downregulation of either target (23, 24, 25). To date, three BsAbs are on the market, and hundreds of others, mostly in the oncology field, are in the preclinical or clinical development stages (26).

Various modular structures of immunoglobulins have been exploited to create alternative BsAb configurations (27, 28), including tetravalent immunoglobulin G-single chain variable fragment (IgG-scFV), which contains a whole mAb-like molecule targeting one antigen, and a tandem C-terminal-attached scFV targeting a different antigen or epitope (28). ScFVs are usually assembled from the antigen-binding domains of the variable heavy (VH) and light chain (VL) regions of mAbs or screened directly out from antibody scFV phage display libraries (29). The symmetric structure of IgG-scFV can overcome heavy and light-chain-pairing problems (30), but inclusion of the scFV may affect the affinity, stability, and solubility of BsAb molecules (31), potentially resulting in loss of activity (32).

Two BsAbs targeting HER2 and PD-1 have been reported and show promising antitumor activities in animal models (33). However, both molecules adopted unmodified IgG1 as their backbone, which may temper their clinical effectiveness. Although IgG1-triggered effector functions such as ADCC, complement-dependent cytotoxicity (CDC), and antibody-dependent cellular phagocytosis (ADCP) contribute to the antitumor activities by trastuzumab, they can cause collateral damage to PD-1-expressing immune cells, especially tumor-infiltrating T cells.

In this study, we generated a stable HER2/PD-L1 BsAb with complete trastuzumab in IgG1 subclass coupled to humanized anti-PD-L1 scFVs. BsAb exhibited enhanced ADCC to tumor cells when compared with trastuzumab. In the late phase of PBMC-humanized tumor xenograft disease model, the BsAb showed superior tumor inhibition compared with mAb and combination therapies, possibly through the inhibition of HER2 signaling, blockade of PD-L1/PD-1 signaling, and enhanced ADCC. Therefore, simultaneously targeting both HER2 and PD-L1 molecules with BsAb strategy may offer a highly effective management solution for trastuzumab-resistant HER2^+^ carcinomas.

## Results

### HER2/PD-L1 BsAb displayed excellent purity and thermostability

**Figure 1:**
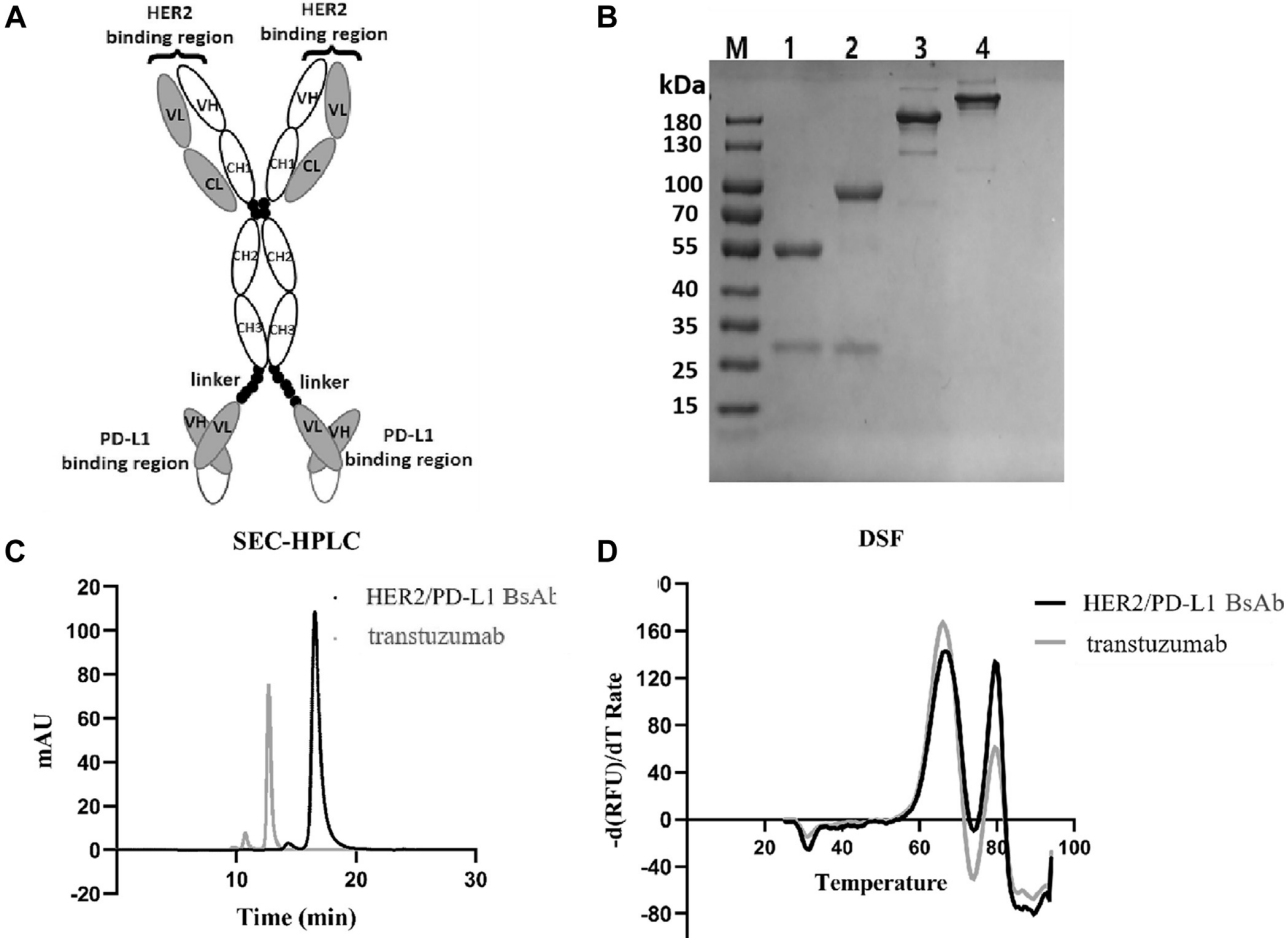
**The structure, purity, and stability of HER2/PD-L1 BsAb.***A*, BsAb is composed of intact trastuzumab IgG1 mAb and scFV fragments from anti-PD-L1 fused to the C-terminal of the trastuzumab Fc *via* a flexible (GGGGS)3 linker. *B*, BsAb and trastuzumab were analyzed by reduced and nonreduced SDS-PAGE. Lane 1, reduced trastuzumab; lane 2, reduced BsAb; lane 3, nonreduced trastuzumab; lane 4, nonreduced BsAb; M, Molecular weight markers. *C*, the purities of BsAb and trastuzumab analyzed by SEC-HPLC. *D*, comparing the thermostabilities of BsAb and trastuzumab by DSF analysis performed on a Bio-Rad real-time PCR instrument.

The HER2/PD-L1 BsAb was constructed with whole trastuzumab IgG1 and tandem anti-PD-L1 scFVs in the format of IgG-scFV (Fig. 1*A*). Following expression and purification, BsAb was evaluated by sodium dodecyl sulfate–polyacrylamide gel electrophoresis (SDS-PAGE), size exclusion chromatography (SEC)-HPLC, and differential scanning fluorimetry (DSF). BsAb displayed excellent purity and thermal stability in these assays (Fig. 1, *B*–*D*).

### Binding and neutralizing activities of BsAb

**Figure 2:**
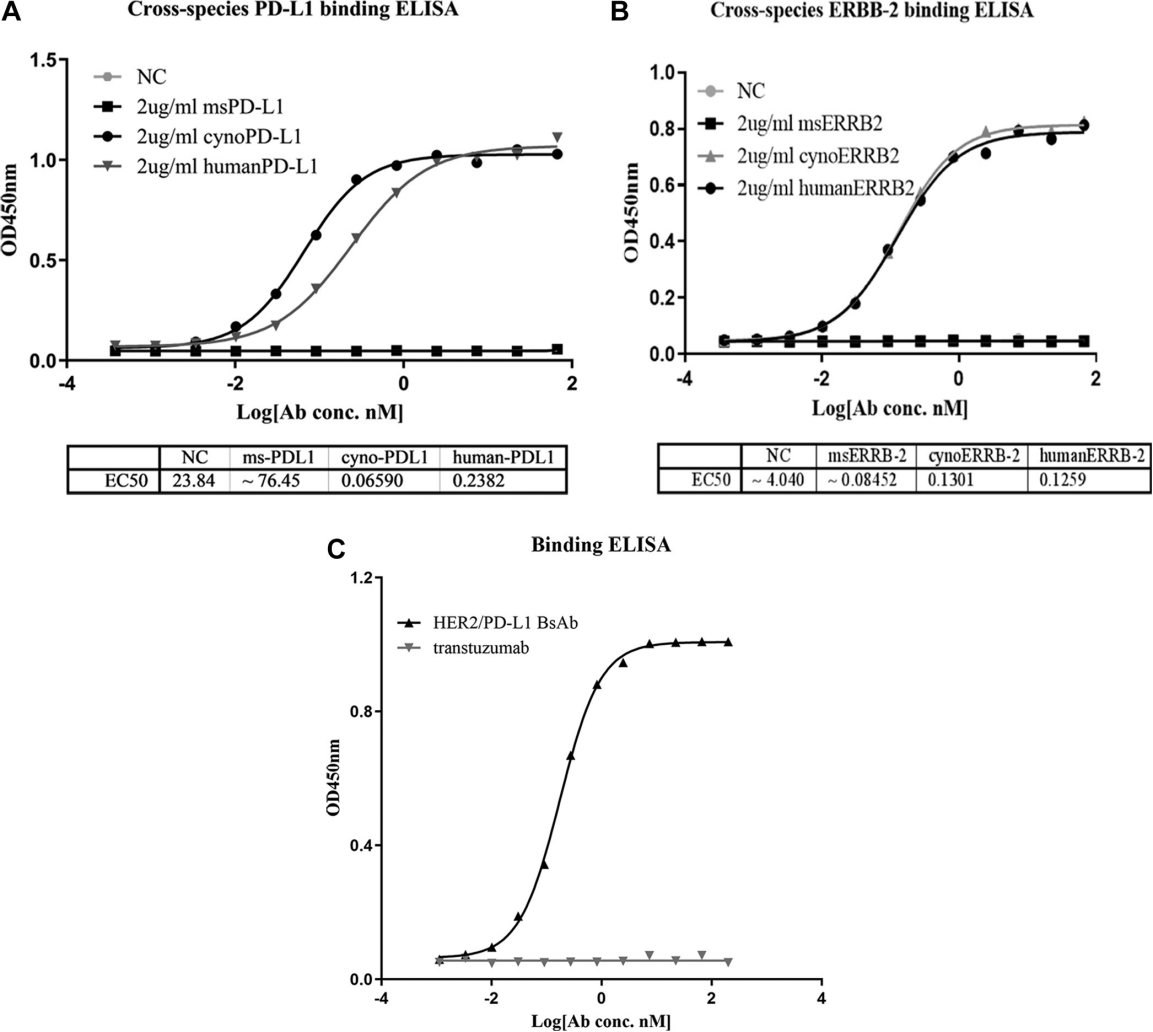
**The binding activities of HER2/PD-L1 BsAb to PD-1 and ERBB-2 from different species.***A*, the binding affinities of HER2/PD-L1 BsAb for mouse (msPD-L1), cynomolgus monkey (cynoPD-L1), and human (humanPD-L1) PD-L1 were measured by ELISA. *B*, the binding affinities of BsAb for mouse (msERBB-2), cynomolgus monkey (cynoERBB-2), and human (humanERBB-2 or HER2) were measured by ELISA. *C*, HER2/PD-L1 could bind human PD-L1 and HER2 simultaneously as determined by bridging ELISA, in which HER2 proteins were coated onto the plates and biotinylated-PD-L1 proteins were used as detection agent.

Firstly, we evaluated the binding properties of BsAb to human, mouse, and cynomolgus ERBB-2 and PD-L1 molecules. We observed dose-dependent binding of BsAb to human and cynomolgus ERBB-2 and PD-L1 but not to mouse ERBB-2 or PD-L1 (Fig. 2, *A* and *B*). To test whether BsAb can simultaneously bind to both targets, bridging ELISA was performed, with the results indicating that BsAb, but not trastuzumab, was capable of binding to HER2 and PD-L1 at the same time (Fig. 2*C*).

We further characterized the binding abilities of BsAb to HER2 and PD-L1 using biolayer interferometry (BLI) technology (Table S1). The equilibrium dissociation constant (K_D_) values of BsAb and trastuzumab binding to HER2 were 6.36 and 7.86 nM, respectively. BsAb’s K_D_ value of binding to human PD-L1 was greater than that of avelumab.

**Figure 3:**
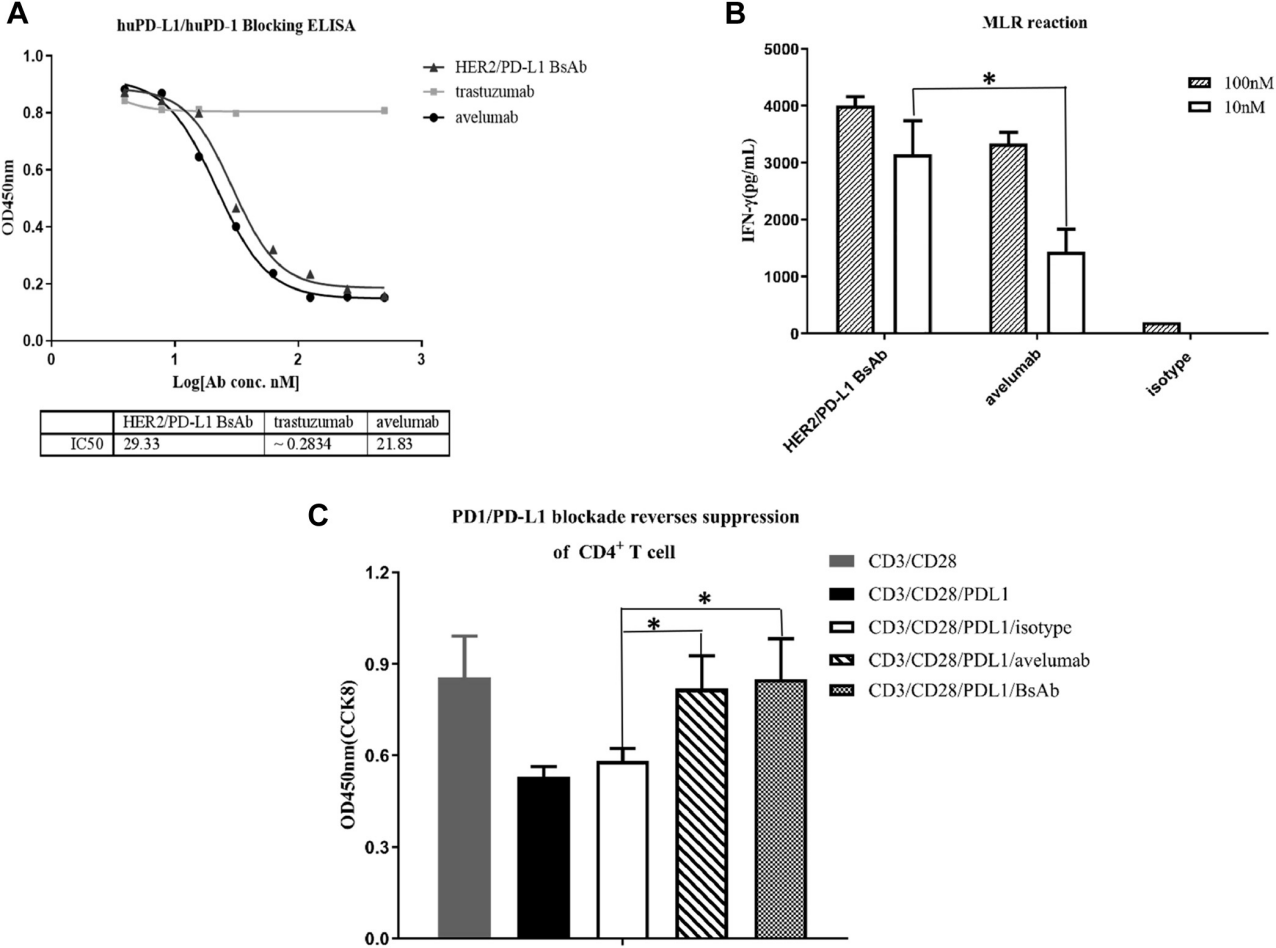
**HER2/PD-L1 BsAb blocked PD1/PD-L1 interaction.***A*, BsAb suppressed PD-L1 interaction with PD-1 in ELISA. Avelumab and trastuzumab were used as positive and negative controls, respectively. *B*, IFN-γ secretion induced by BsAb in mixed lymphocyte reaction (MLR) assays. CD4^+^ T cells were cocultured with allogeneic DCs in the presence of BsAb, avelumab, or isotype for 6 days. Supernatants were harvested on day 5 to measure IFN-γ secretion by ELISA. *C*, BsAb overcame PD-L1-mediated suppression of T-cell activation. The effects of BsAb treatment on IFN-γ secretion in primary T cell activation induced by anti-CD3, anti-CD28 and PD-L1. ∗*p* < 0.05 as indicated by Student’s *t* test. Data were represented as mean ± STE.

Neutralizing ELISA was conducted to assess the ability of BsAb to block PD-L1 and PD-1 interaction (Fig. 3*A*). Both BsAb and avelumab efficiently blocked the binding of human PD-1 to PD-L1 at relatively low concentrations, with *IC50* values of 29.33 and 21.83 nM, respectively.

### Activation of T cells *in vitro*

Mixed lymphocyte reaction (MLR) was utilized first to measure the activities of BsAb at activating T cells. Mature dendritic cells were cocultured with T cells from different donors to stimulate allogenic T cell activation and subsequent release of cytokines, such as IFN-γ. Both BsAb and avelumab markedly augmented IFN-γ production, but the former was more potent than the latter at concentrations of both 10 and 100 nM (Fig. 3*B*), even though a significant difference was not observed at 100 nM. Anti-CD3 and anti-CD28-stimulated human CD4^+^ T cell proliferation assay was also used to further examine BsAb at the cellular level. As shown in Figure 3*C*, T cell proliferation was significantly reduced by incubation with PD-L1. BsAb and avelumab, but not the isotype control treatment, reversed the inhibitive effects of PD-L1 (Fig. 3*C*). Therefore, BsAb retained and, under certain circumstances, enhanced the ability of PD-L1 mAb to activate T cells in two different cell-based assays.

### Antitumor activities *in vitro*

**Figure 4:**
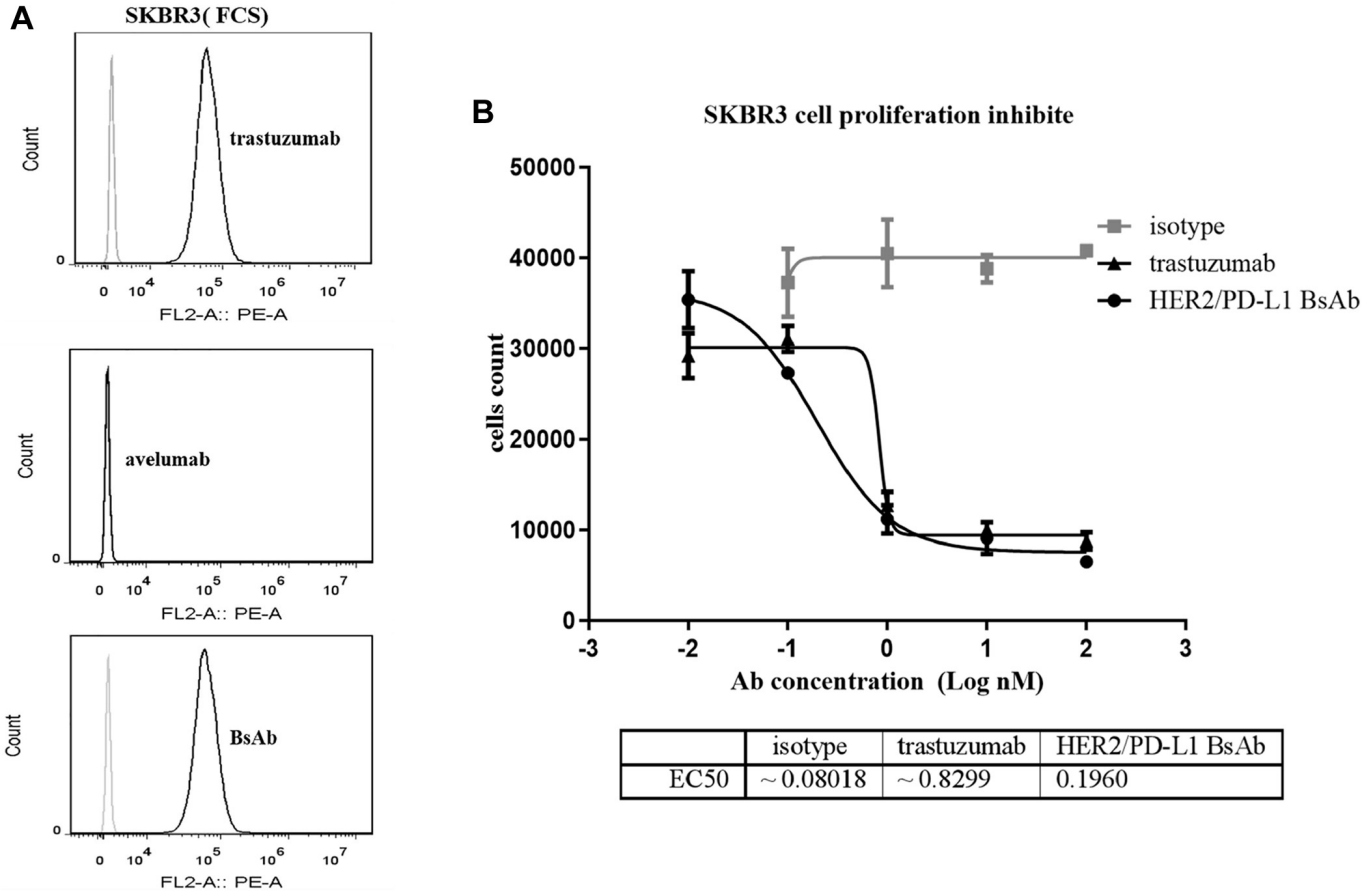
**HER2/PD-L1 BsAb suppressed the proliferation of HER2**^**+**^**tumor cells.***A*, HER2/PD-L1 BsAb and trastuzumab, but not avelumab, could bind to SKBR3 cells. *B*, HER2/PD-L1 BsAb and trastuzumab, but not isotype control, suppressed SKBR3 cells proliferation in CCK-8 assay.

We first utilized SKBR3 cells to examine the ability of BsAb to directly inhibit HER2^+^ tumor cells *in vitro*. Both BsAb and trastuzumab were effective in inhibiting SKBR3 cell proliferation (Fig. 4*B*), and the half maximal inhibitory concentration (*IC*_*50*_) values were determined to be 0.1960 and 0.8229 nM, respectively.

**Figure 5:**
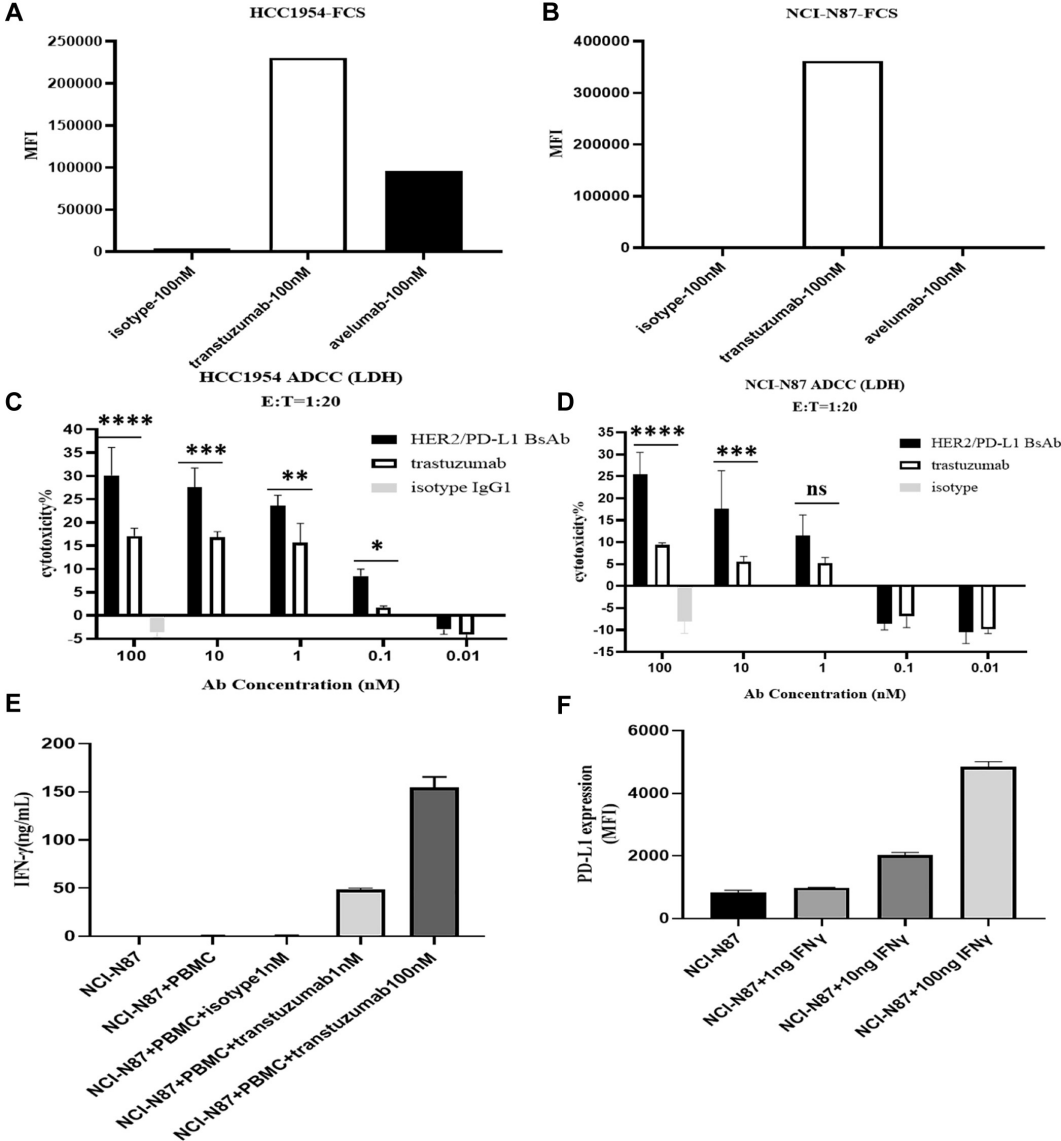
**The enhanced ADCC activities of HER2/PD-L1 BsAb.***A* and *B*, HER2 and PD-L1 expression levels on HCC1954 and NCI-N87 cells were measured by flow cytometry. *C* and *D*, ADCC activities of HER2/PD-L1 BsAb and trastuzumab to HCC1954 and NCI-N87 cells using human PBMCs as effector cells. Tumor cells were cocultured with human PBMCs (1:20) in the presence of HER2/PD-L1 BsAb, trastuzumab, or isotype for 18 h. Supernatants were harvested to measure the LDH release by ELISA. *E*, the production of IFN-γ in tumor cells and human PBMC coculture was measured by ELISA. *F*, IFN-γ-induced upregulation of PD-L1 expression on NCI-N87 cells as determined by flow cytometry.∗*p* < 0.05, ∗∗*p* < 0.01, ∗∗∗*p* < 0.001, and ∗∗∗∗*p* < 0.0001 as indicated by Student’s *t* test. Data were represented as mean ± STE.

One of the key mechanisms of action (MOAs) of trastuzumab is ADCC (34), and as such, we compared the abilities of BsAb and trastuzumab in promoting ADCC using human PBMCs as effector cells and HER2^+^ NCI-N87 and HCC1954 tumor cells as target cells. Interestingly, BsAb exhibited much more efficient ADCC than trastuzumab in both cell lines, even though NCI-N87 cells did not initially express PD-L1 (Fig. 5, *A* and *B*). Nevertheless, we found that trastuzumab treatment could upregulate IFN-γ production in the supernatant of the coculture of PBMCs with either tumor cell line (Fig. 5*E*), and IFN-γ treatment could elevate the expression of PD-L1 in both HCC1954 and NCI-N87 cells (Fig. 5*F*). Taken together, our data suggest that the HER2/PD-L1 BsAb might enhance trastuzumab-mediated ADCC through IFN-γ-mediated upregulation of PD-L1 expression in both PD-L1^+^ and PD-L1^−^ tumor cells.

### Antitumor activities in PBMC-“humanized” tumor xenograft models

Since BsAb does not cross-react with mouse PD-L1, we injected human peripheral blood mononuclear cells (PBMCs) into immunocompromised NOD SCID gamma (NSG) mice to create PBMC-“humanized” immune system in mice, on which tumor xenograft model was established.

**Figure 6:**
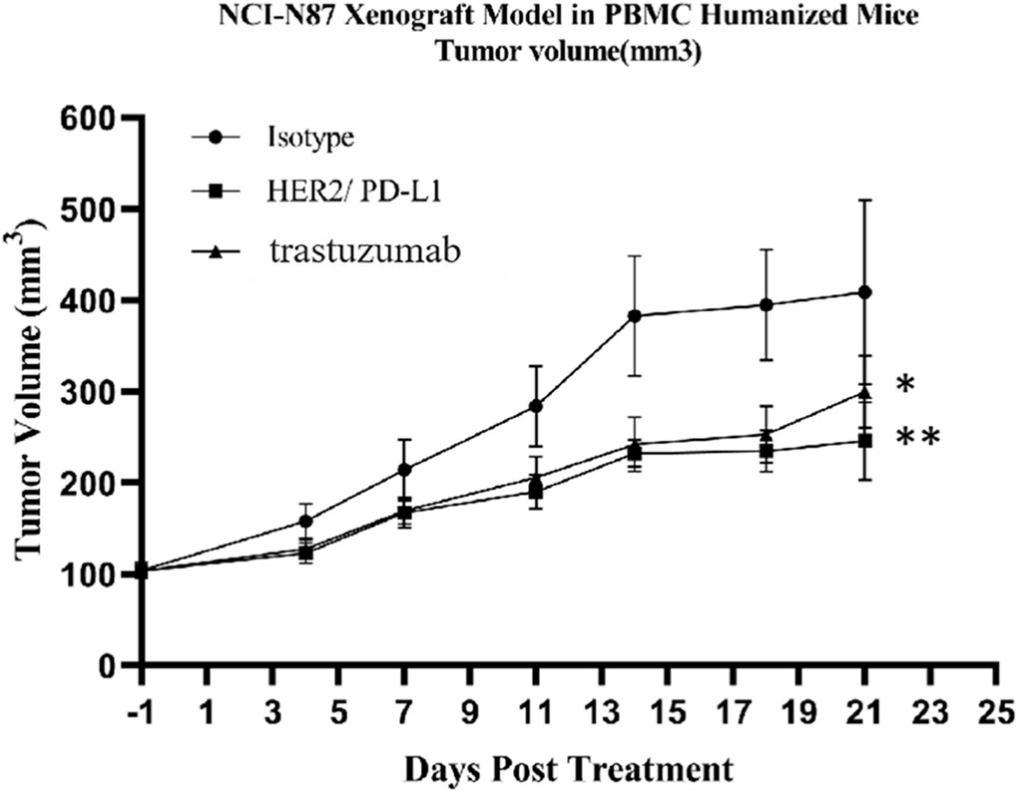
**Antitumor responses of HER2/PD-L1 BsAb in NCI-N87 tumor xenograft model.** NOG mice were subcutaneously inoculated with NCI-N87 cells and human PBMCs (10^7^ cells/mouse) were injected to the tumor sites when tumor volumes reached 100 mm^3^. Mice were randomized into different groups and received i.v. administration of BsAb (10 mg/kg, n = 8), trastuzumab (7.5 mg/kg, n = 8) or isotype control (7.5 mg/kg, n = 8), BIW, as indicated. ∗*p* < 0.05 and ∗∗*p* < 0.01 as indicated by one-way ANOVA followed by Newman–Keuls multiple comparisons test. Data were represented as mean ± STE.

Firstly, we compared the antitumor activities of BsAb and trastuzumab in the HER2^+^PD-L1^−^ NCI-N87 tumor xenograft model. Both BsAb and trastuzumab exhibited significant inhibition of tumor growth. Interestingly, BsAb displayed comparable antitumor activities to trastuzumab in the early phase of the model but gradually gained advantage over the latter in the late phase of the disease model (Fig. 6).

**Figure 7:**
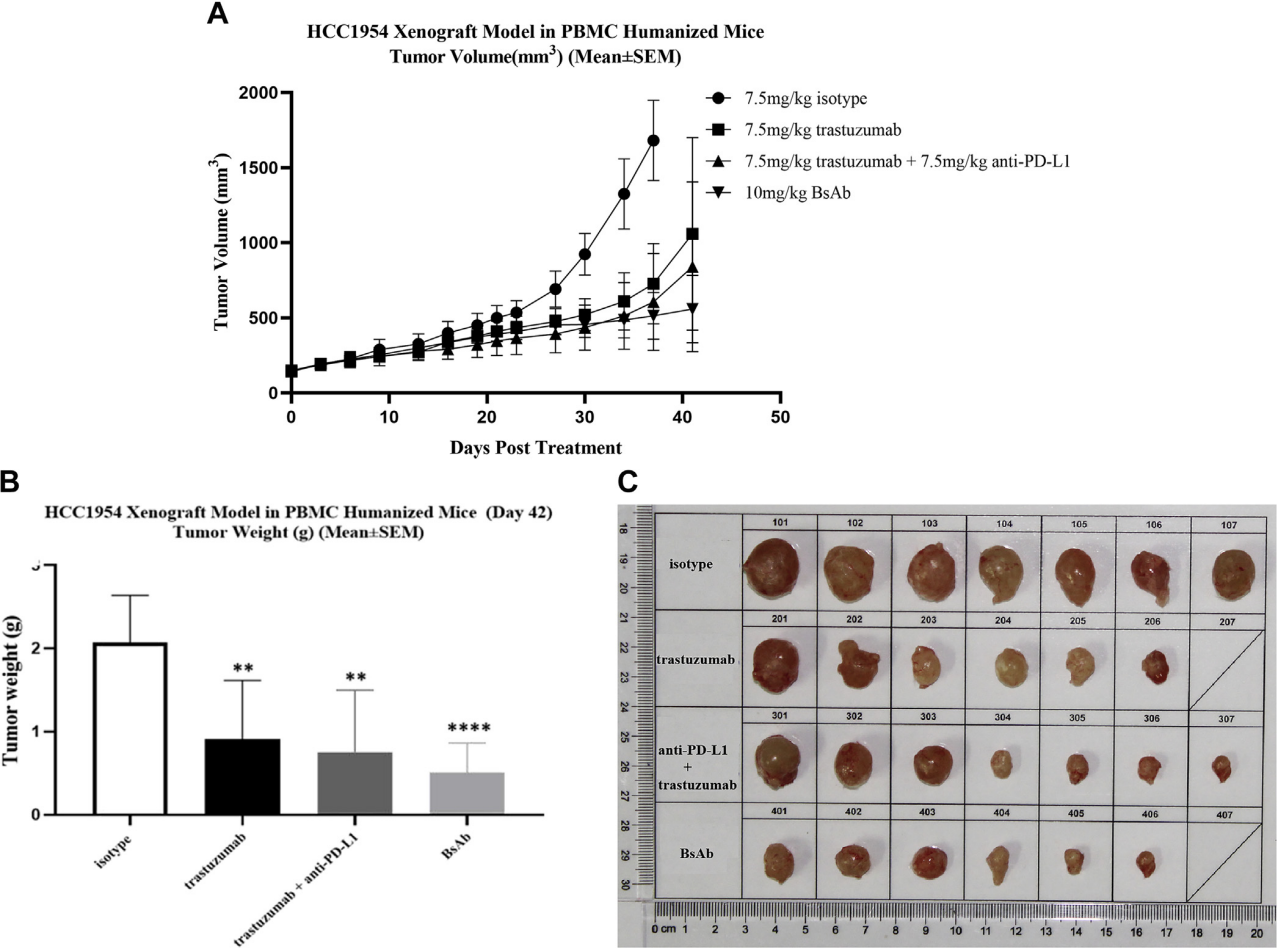
**HER2/PD-L1 BsAb was superior to mAb and combination therapies in PBMC-“humanized” HCC1954 xenograft model.** NOG mice were subcutaneously inoculated with HCC1954 cells and human PBMCs (10^7^ cells/mouse) were injected to the tumor sites when tumor volumes reached 100 mm^3^. NOG mice were randomized into different groups after mean tumor volumes reached 150 mm^3^ and received i.v. administration of BsAb (10 mg/kg, n = 7, G4), trastuzumab (7.5 mg/kg, n = 7, G2), trastuzumab + anti-PD-L1 mAb (7.5 mg/kg +7.5 mg/kg, n = 7, G3), or isotype control (7.5 mg/kg, n = 7, G1), BIW, as indicated. *A*, tumor growth in PBMC-“humanized” HCC1954 xenograft model after received different treatments. *B*, tumor weights on the day when the group of mice were euthanized. *C*, tumor images on the day when the group of mice were euthanized. ∗∗*p* < 0.01 and ∗∗∗*p* < 0.001 as indicated by one-way ANOVA followed by Newman–Keuls multiple comparisons test. Data were represented as mean ± STE.

Secondly, we analyzed the antitumor activities of BsAb in HER2 and PD-L1 double-positive PBMC-“humanized” HCC1954 tumor xenograft mouse model. BsAb exhibited significantly stronger tumor growth inhibition (TGI: 71.36%) in comparison to trastuzumab (TGI: 56.17%) and combination therapy (TGI: 63.55%) on day 41 (Fig. 7, *B* and *C*, and Table S2). Similar to the NCI-N87 xenograft model, there was no significant difference among BsAb, trastuzumab, and combination treatments in the early phase of the model, but the BsAb treatment group gradually gained superiority (Fig. 7*A*) and became the only group to achieve *p* < 0.0001 in tumor volume in comparison to the isotype-treated group (Fig. 7*B*). No significant changes in body weight or toxicity were observed among any group (data not shown).

Therefore, our *in vivo* results suggest that BsAb is superior to trastuzumab or even the combination therapy in battling HER2-expressing, PD-L1 positive or negative, human tumors.

### Favorable PK and toxicity profiles in cynomolgus monkeys

**Figure 8:**
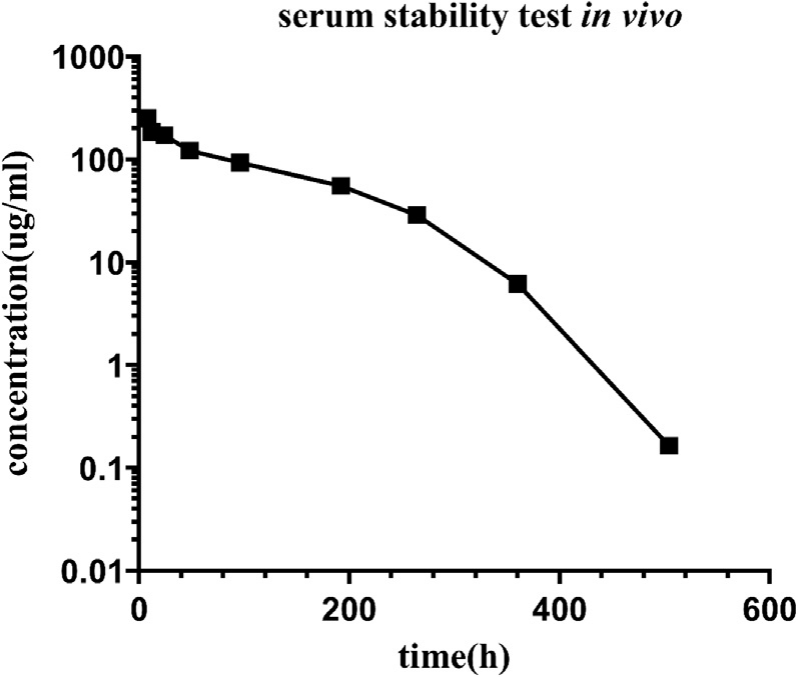
**Serum concentration of intact HER2/PD-L1 BsAb after a single i.v. administration of 10 mg/kg HER2/PD-L1 BsAb to cynomolgus monkeys.** n = 2 monkeys per time point. Serum concentrations were measured with antigen capture ELISA.

BsAb was administered (10 mg/kg single intravenous injection) into cynomolgus monkeys, blood samples were collected at set time points, and serum concentrations of intact BsAb were measured by ELISA. BsAb exhibited the typical PK profile of antibodies (Fig. 8), and its serum concentration was still detected at the 504-h time point. In addition, no blood, liver, or renal toxicities of BsAb were observed in two cynomolgus monkeys (one male and one female), as revealed by blood biochemistry and cell tests (Fig. S1).

## Discussion

Trastuzumab has been tested in patients with various solid tumors harboring HER2 overexpression, amplifications, and/or mutations (35, 36). Unfortunately, its successes in BCs and GCs have not been recapitulated in other HER2^+^ cancer types such as BTC, CRC, NSCLC, or bladder cancers (37, 38). The diverse responses of solid tumors to trastuzumab may be due to the difference in the levels of HER2 expression, degrees of dependence, and/or other aspects of the disease biology. Although a variety of anti-HER2 therapeutics are being actively pursued, including antibody–drug conjugates (39, 40) and BsAbs, such as HER2/HER2 (different epitopes), HER2/HER3, and HER2/CD3 BsAbs (41, 42, 43, 44), these therapeutic strategies are not expected to completely overcome induced or intrinsic drug resistance to anti-HER2 therapies.

A number of studies have demonstrated that HER2-targeted therapies can induce upregulation of PD-L1 expression in HER2^+^ tumors (45). Previous studies have reported that the combination of trastuzumab and pembrolizumab exhibits long-lasting clinical benefits in HER2^+^ BC, GEJC, GC, and esophageal cancers, reinforcing the hypothesis that upregulation of PD-L1 contributes to resistance to trastuzumab (20, 22). In our study, we constructed a HER2/PD-L1 BsAb in the IgG1 subclass with the purpose of combining HER2 targeted therapy with immune checkpoint PD-1/PD-L1 blocking therapy to treat HER2-overexpressing solid tumors. We mimicked the trastuzumab-induced clinically observed drug resistance with the late phase of PBMC-humanized HCC1954 xenograft tumor model. HER2/PD-L1 BsAb demonstrated advantages over mAb and combination therapies, achieving synergy between targeting HER2 and PD-L1 pathways. Therefore, HER2/PD-L1 BsAbs are applicable to treat solid tumors expressing both HER2 and PD-L1 and to overcome tumor resistance to HER2-directed therapies clinically.

Two HER2/PD-1 BsAbs, both in the IgG1 subclass, have been reported, with one entering Phase I clinical trial (NCT04162327) (33). HER2/PD-L1 BsAbs provide an MOA different from that of HER2/PD-1 BsAbs and may offer patients additional clinical benefits. IgG1 in trastuzumab is a potent trigger of ADCC and CDC (46), mediated by NK cells and complements to attack HER2^+^ tumor cells. In the case of HER2/PD-1 BsAb, however, IgG1 may cause collateral damage to activated T cells. Although HER2/PD-1 BsAbs in IgG2 or IgG4 can spare damages to activated T cells, they are unable to engage tumor-targeted ADCC and CDC. This dilemma could be potentially solved by using HER2/PD-L1 BsAbs in the IgG1 subclass, since PD-L1 is mainly expressed in tumor cells. The caveat of this strategy is that HER2/PD-L1 BsAbs cannot bridge PD-1-expressing T cells to HER2-expressing tumors. Because human NK cells cannot be reconstructed in the PBMC-“humanized” tumor xenograft model, the question of whether the antitumor efficacy and potency of HER2/PD-L1 in IgG1 are superior to that of HER2/PD-1 BsAb in IgG4, which is currently under intense testing at our laboratory, is yet to be answered.

ADCC is one of the primary MOAs of trastuzumab. Interestingly, HER2/PD-L1 BsAb displayed more efficient ADCC than trastuzumab to not only PD-L1^+^ but also to PD-L1^−^ tumor cells. We questioned how this enhanced efficiency was achieved. Chaganty *et al.* (8) previously reported that targeting HER2 with trastuzumab could upregulate PD-L1 expression in tumor cells by inducing IFN-γ production in the tumor microenvironment, thereby contributing to induced immune resistance to HER2 targeted therapy. In our study, we demonstrated that trastuzumab induced the release of IFN-γ in the coculture of PBMCs with both N87 and HCC1954 tumor cell lines. IFN-γ production, in turn, upregulated the level of PD-L1 expression in both tumor cell lines that were originally PD-L1-negative. As a consequence, the ADCC effect of BsAb was enhanced because of the upregulation of PD-L1 on the tumor cell surface. Thus, we suggest that during trastuzumab treatment in cancer patients, trastuzumab stimulates IFN-γ production, which turns PD-L1-negative “hot” tumors into PD-L1^+^ “cold” tumors, evading immune attacks mediated by T and NK cells. Our preclinical results indicated that the application of HER2/PD-L1 BsAb could potentially prevent this “turning” process and thus achieved long-lasting clinical benefits in our tumor xenograft models.

In summary, HER2/PD-L1 BsAb in the IgG1 subclass retained the activities of both parental mAbs and demonstrated enhanced ADCC to HER2^+^ tumor cells. In the late stage of the PBMC-“humanized” HCC1954 xenograft tumor model, its therapeutic efficacy was superior to mAb and combination therapies. Collectively, HER2/PD-L1 BsAb was potentially an effective option for managing HER2^+^ but trastuzumab-resistant tumors.

## Experimental procedures

### Cell lines and reagents

NCI-N87, HCC1954, and SKBR3 cells were obtained from the Shanghai Cell Line Bank. RPMI1640 medium (61870036), Freestyle 293 medium, Dynabeads CD4 Positive Isolation Kit (11331D), TMB substrate, and fetal calf serum (FCS) were obtained from Thermo Fisher Scientific. PE-labeled goat anti-human IgG (409304), streptavidin-PE (405203), anti-human-CD3 (clone HIT3a), and anti-human-CD28 (clone CD28.2) were obtained from Biolegend. Trastuzumab and humanized anti-PD-L1 scFV were prepared in house. Cell Counting Kit-8 (CCK8) was obtained from Dojindo Laboratories. Human antibody germline sequence and primers were synthesized by Sangon Biotech. IL-4 and GM-CSF were obtained from R&D Systems, and all other recombinant proteins were products of Sino Biological. SYPRO Orange was obtained from Sigma-Aldrich. Human IFN-γ ELISA Kit (EHC102g) was obtained from Neobioscience. Cytotoxicity detection kit (LDH) was from Roche Life Science. Matrigel was obtained from BD Biosciences. Polyethylenimine (PEI) was obtained from Polysciences. Protein A probes were obtained from Pall Corporation. Protein A-Sepharose Column was obtained from BioVision. HRP-conjugated goat anti-human IgG (H + L) was obtained from Jackson ImmunoResearch. Monocyte purification kit was obtained from Miltenyi Biotec. Ficoll gradient was obtained from GE healthcare. Human PBMCs were obtained from Allcells.

### Humanization of anti-PD-L1 mAb by framework shuffling

Murine hybridoma cells secreting mAbs against human PD-L1 were prepared in a different study. Total RNA was isolated from hybridoma cells, and variable region genes of the PD-L1 antibody were obtained by degenerate polymerase chain reaction (PCR). Anti-PD-L1 mAb was humanized using a framework shuffling strategy with human heavy and light germline genes. The procedure was carried out as previously described (25) and resulted in the discovery of one humanized antibody molecule exhibiting excellent thermal stability, binding affinity, and specificity as determined by ELISA.

### Vector construction, protein expression, and purification

A disulfate bond was inserted into anti-PD-L1 scFVs between VH (heavy chain variable domain) 44 and VL (light chain variable domain) 100 to increase the stability of scFV molecules (24). Trastuzumab light and heavy chain amino acid sequences were obtained from the international ImMunoGeneTics information system (IMGT/mAb-DB). Anti-PD-L1 scFV was linked to the C-terminus of trastuzumab Fc using flexible peptide linker (GGGGS)_3_. The DNA sequences were synthesized and subcloned into a pcDNA3.1 vector and amplified in *E. coli*. Purified plasmids were then transfected into HEK293F cells using the PEI technique. Subsequently, the cells were grown in suspension in Freestyle 293 expression medium. After 6 days of culture, the supernatant was collected, and the antibodies were purified using Protein A columns. The purified antibodies were dialyzed against phosphate buffered saline (PBS), concentrated, flash frozen, and kept at −8 °C.

### Binding ELISA

The potency of BsAb interaction with antigens (PD-L1 and HER2) derived from different species was assessed using enzyme-linked immunosorbent assay (ELISA). Ninety-six-well immuno-plates were coated with 2 μg/ml 6xHis-tagged antigen proteins overnight at 4 °C. Three-fold serial dilutions of BsAb and control antibodies (avelumab and trastuzumab) were applied to wells. Following 1-h incubation, horseradish peroxidase (HRP)-conjugated goat anti-human IgG (H + L) was added and incubated for 1 h at 37 °C. The plate was then washed and developed with 3,3′,5,5′-tetramethylbenzidine (TMB) substrate. Visualization was performed at 450 nm using a SpectraMax M5e (Molecular Devices) microplate reader.

### Dual-target bridging ELISA

PD-L1-ECD protein (2 μg/ml) was coated onto 96-well immuno-plates (Greiner) at 4 °C overnight. The plates were washed with PBS, blocked for 1 h with 1% casein in PBS, and incubated with three-fold serial dilutions of antibodies for another hour at 37 °C. The plates were then washed three times and incubated with 2 μg/ml biotinylated-HER2 protein for 1 h at 37 °C. After washing, streptavidin-HRP was added and incubated for 1 h at 37 °C. The plates were washed and the reaction developed with TMB substrates. Visualization was performed at 450 nm using a SpectraMax M5e microplate reader.

### Biolayer interferometry (BLI)

BsAbs were diluted to 10 μg/ml using sample buffer (0.02% Tween 20 and 0.1% BSA in PBS). Hu (human)-HER2 and hu-PD-L1 proteins (6xHis-tagged) were diluted to 100 nM using sample buffer. Binding of BsAb with specific hu-HER2 and hu-PD-L1 antigens was analyzed on ForteBio’s Octet RED96 BLI system. All measurements were performed in the sample buffer at room temperature (RT). BsAb (10 μg/ml) was immobilized onto Protein A biosensors, and antigens were twofold serially titrated from 100 nM to achieve rate constants and affinity measurements. *KD* values were calculated using the vendor-supplied software.

### PD1/PD-L1 blocking ELISA

The ability of BsAb to block the PD1 and PD-L1 interaction was measured using a competitive ELISA. In brief, hu-PD-L1 protein was coated onto 96-well immuno-plates (Greiner) at 4 °C overnight. Next, threefold serial dilutions of BsAb and trastuzumab were introduced to the wells along with 1 μg/ml of biotinylated hu-PD1 protein. Following a 1-h incubation, the bound competitive ligands were detected using HRP-conjugated streptavidin and developed with TMB substrate. The *IC*_*50*_ of antibodies was measured using software on a SpectraMax M5e microplate reader.

### Flow cytometry

HER2 and PD-L1 expression levels in HCC1954 and NCI-N87 human cancer cells were measured by flow cytometry. In short, single-cell suspensions (3 × 10^5^ cells) were stained with avelumab (for PD-L1), trastuzumab (for HER2), and isotype-matched control in 5-ml FACS tubes. After washing, the cells were incubated with PE-conjugated anti-human IgG antibody and then analyzed by a CytoFLEX flow cytometer (Beckman Coulter). To measure IFN-γ-induced PD-L1 expression levels, tumor cells were split into 6-well cell culture plates at 4 × 10^5^ cells/well and cultured with different concentrations of IFN-γ (1, 10, or 100 ng/ml) for 24 h at 37 °C in complete media. Single cancer cell suspensions (4 × 10^5^ cell/ml) were then prepared, and the expression levels of PD-L1 were determined by flow cytometry as described above.

### Differential scanning fluorimetry (DSF)

DSF was used to evaluate the thermal stability of antibodies. One microliter of SYPRO Orange (#S6650, Thermo Fisher Scientific) 250× working solution was added into 24 μl of PBS-diluted antibody at 20 μM. Relative fluorescence units (RFUs), dRFU/dT curve, and primer melting temperature (*Tm*) values were obtained on a CF×96 Touch qPCR machine (Bio-Rad).

### Size exclusion chromatography (SEC)-HPLC

SEC-HPLC analysis was conducted to evaluate the purity, aggregation, and degradation of antibodies. Samples were analyzed with an Agilent 1260 HPLC system (Agilent) on a Thermo MAbPac SEC-1, 5 μm, (7.8 mm × 300 mm) column (P/N 088460). Mobile-phase condition was 50 mM sodium phosphate, 300 mM sodium chloride, pH 6.8. SEC chromatograms were observed at 280-nm absorbance at 25 °C, with a flow rate of 0.7 ml/min and sample injection volume of 15 μl.

### Mixed lymphocyte reaction (MLR)

Human blood monocytes were extracted from human PBMCs with a monocyte purification kit and grown in culture for 7 days in 500 U/ml IL-4 and 250 U/ml GM-CSF to produce dendritic cells (DCs). Subsequently, CD4^+^ T cells (1 × 10^5^ cells) and allogeneic DCs (1 × 10^4^ cells) were cocultured, in the presence or absence of BsAb, avelumab, or isotype-matched control. After 5 days of culture, cell supernatants were collected to measure secreted IFN-γ with ELISA.

### T-cell activation assay

Anti-CD3 (clone HIT3a, 1 μg/ml), anti-CD28 (clone CD28.2, 1 μg/ml), and human PD-L1 (6xHis-tagged, 5 μg/ml) were coated onto Corning 96-well cell culture plates overnight at 4 °C. Control wells were coated with either mouse IgG2a isotype control alone or with anti-CD3 and anti-CD28 antibodies. CD4^+^ T cells were extracted with Dynabeads CD4 cell positive isolation kit according to manufacturer’s guidelines. CD4^+^ T cells were cultured in precoated 96-well plates, in the presence or absence of antibodies at the abovementioned concentrations for 4 days at 37 °C in RPMI1640 with 10% FBS. After 4 days, cells were counted using a CCK-8 kit.

### Tumor cell inhibition

Tumor cells were split into 96-well plates at 10,000 cells/well. After incubation overnight, serial dilutions of BsAb and trastuzumab in culture media were added to the wells. The plates were maintained in a CO_2_ incubator for 96 h at 37 °C. Subsequently, CCK-8 agent was introduced, and the cells were further incubated until untreated control cells’ OD450 values were greater than 1. Growth suppression was evaluated as the percentage of growth relative to untreated cells. The dose–response curves were plotted from the average of three measurements. *IC50* values were calculated using software on a SpectraMax M5e microplate reader.

### Antibody-dependent cellular cytotoxicity (ADCC)

Human PBMCs were purified from the “buffy coats” provided by Zhongshan Blood Center with Ficoll gradient according to manufacturer’s guidance. NK cells were extracted from human PBMCs with negative selection and magnetic beads (Miltenyi Biotec). NK cells (3 × 10^6^ cells) and target cells (3 × 10^5^ cells) were cocultured in the presence or absence of testing antibodies. Following incubation for 18 h, LDH release into culture supernatants was analyzed by ELISA. After an additional 18-h incubation, the cell supernatant was collected to measure the concentrations of secreted IFN-γ by ELISA.

### Antitumor effect in PBMC-“humanized” tumor xenograft models

HCC1954 cells (5 × 10^6^ cells) were subcutaneously administered to the right flank of NOG mice on day 12. Next, PBMCs (1 × 10^7^ cells) were resuspended with PBS and administered *via* the tail veins of NOG mice on day 7. The mean tumor volume was ∼100 mm^3^ 12 days post inoculation (day 0). At this time point, the mice were assigned to four equal groups based on their tumor volumes. Mice were dosed intraperitoneally (IP) as listed below: Group 1, 7.5 mg/kg isotype; Group 2, 7.5 mg/kg trastuzumab; Group 3, 7.5 mg/kg trastuzumab +7.5 mg/kg anti-PD-L1 mAb; Group 4, 10 mg/kg BsAb. Administrations were routinely performed on days 0, 4, 7, 11, 14, 18, 21, 29, and 37 post inoculation. Tumor volume was measured twice a week and calculated as *V = LW*^*2*^*/2* (*V* = volume, *L* = length, *W* = width).

Similarly, NCI-N87 cells (5 × 10^6^ cells) were subcutaneously administered to the right flank of NOG mice on day 14. PBMCs (5.5 × 10^6^ cells) resuspended in PBS were administered to the tail veins of NOG mice on day 9. The mean tumor volume was ∼100 mm^3^ 14 days post inoculation (day 0). At this time, the mice were equally assigned to three groups based on their tumor volumes: Group 1, 7.5 mg/kg isotype; Group 2, 10 mg/kg BsAb; and Group 3, 7.5 mg/kg trastuzumab. Administrations were routinely performed IP on days 0, 4, 7, 11, 14, and 18 post inoculation. Tumor volumes were measured and calculated as described above. All live animal studies were approved by the Ethical Committee of SIMM, CAS.

### Serum stability in cynomolgus monkeys

In a single-dose serum stability examination, two cynomolgus monkeys were injected intravenously with 10 mg/kg BsAb. Subsequently, blood samples were collected in anticoagulant tubes at 0, 1, 4, 8, 12, 24, 48, 96, 192, 264, 360, and 504 h post administration. Concentrations of BsAb in cynomolgus monkey sera were measured using the standard curve plotted by ELISA. Hu-PD-L1-6xHis was coated onto plates (Greiner) overnight, washed, and incubated with serial dilutions of monkey sera collected. After washing, biotinylated HER2 protein was added and incubated for 1 h. HRP-streptavidin was then developed with TMB substrate. The OD450 value was measured using a SpectraMax M5e microplate reader. Blood samples were collected on days 0, 8, 18, and 22 post administration for toxicity tests with a Cobasb 221 system (Roche) and an automated hematology analyzer (SYSMEX). All live animal studies were approved by the Ethical Committee of SIMM, CAS.

### Statistical analysis

For comparison between two groups, Student’s *t* test was used. For multiple group comparison, one-way ANOVA followed by Newman–Keuls multiple comparisons test was used. In both cases, *p* value less than 0.05 was considered significant. Data are represented as mean ± STE in our results.

## Data availability

All the data are within the manuscript and supporting information. All the data are to be shared upon request (Yi-Li Chen, Fudan University, cheny@dartsbio.com).

## Supporting information

This article contains supporting information.

## Conflict of interest

Y.-L. C., Y. C., X. L., and X. D. received stipends from Shanghai Mabstone Biotechnology, Ltd. G. L. and C. W. are employees of Dartsbio Pharmaceuticals Ltd.

## References

[1] Harbeck N., Pegram M.D., Ruschoff J., Mobus V. (2010). Targeted therapy in metastatic breast cancer: The HER2/neu oncogene. Breast Care (Basel).

[2] Li Q., Lv M., Jiang H., Wang Y., Yu S., Li W., Yu Y., Liu T. (2020). A prospective observational study on the optimal maintenance strategy in HER2-positive advanced gastric cancer treated with trastuzumab-based therapy. J. Cancer Res. Clin. Oncol..

[3] Macpherson I.R., Spiliopoulou P., Rafii S., Saggese M., Baird R.D., Garcia-Corbacho J., Italiano A., Bonneterre J., Campone M., Cresti N., Posner J., Takeda Y., Arimura A., Spicer J. (2019). A phase I/II study of epertinib plus trastuzumab with or without chemotherapy in patients with HER2-positive metastatic breast cancer. Breast Cancer Res..

[4] Cameron D., Piccart-Gebhart M.J., Gelber R.D., Procter M., Goldhirsch A., de Azambuja E., Castro G., Untch M., Smith I., Gianni L., Baselga J., Al-Sakaff N., Lauer S., McFadden E., Leyland-Jones B. (2017). 11 Years' follow-up of trastuzumab after adjuvant chemotherapy in HER2-positive early breast cancer: Final analysis of the HERceptin adjuvant (HERA) trial. Lancet.

[5] He L., Du Z., Xiong X., Ma H., Zhu Z., Gao H., Cao J., Li T., Li H., Yang K., Chen G., Richer J.K., Gu H. (2017). Targeting androgen receptor in treating HER2 positive breast cancer. Sci. Rep..

[6] Rexer B.N., Arteaga C.L. (2012). Intrinsic and acquired resistance to HER2-targeted therapies in HER2 gene-amplified breast cancer: Mechanisms and clinical implications. Crit. Rev. Oncog..

[7] Valabrega G., Montemurro F., Aglietta M. (2007). Trastuzumab: Mechanism of action, resistance and future perspectives in HER2-overexpressing breast cancer. Ann. Oncol..

[8] Chaganty B.K.R., Qiu S., Gest A., Lu Y., Ivan C., Calin G.A., Weiner L.M., Fan Z. (2018). Trastuzumab upregulates PD-L1 as a potential mechanism of trastuzumab resistance through engagement of immune effector cells and stimulation of IFNgamma secretion. Cancer Lett..

[9] Turcotte M., Allard D., Mittal D., Bareche Y., Buisseret L., Jose V., Pommey S., Delisle V., Loi S., Joensuu H., Kellokumpu-Lehtinen P.L., Sotiriou C., Smyth M.J., Stagg J. (2017). CD73 promotes resistance to HER2/ErbB2 antibody therapy. Cancer Res..

[10] Blank C., Gajewski T.F., Mackensen A. (2005). Interaction of PD-L1 on tumor cells with PD-1 on tumor-specific T cells as a mechanism of immune evasion: Implications for tumor immunotherapy. Cancer Immunol. Immunother..

[11] Wherry E.J. (2011). T cell exhaustion. Nat. Immunol..

[12] Zou W., Chen L. (2008). Inhibitory B7-family molecules in the tumour microenvironment. Nat. Rev. Immunol..

[13] Gibbons R.M., Liu X., Pulko V., Harrington S.M., Krco C.J., Kwon E.D., Dong H. (2012). B7-H1 limits the entry of effector CD8(+) T cells to the memory pool by upregulating Bim. Oncoimmunology.

[14] Varadan V., Gilmore H., Miskimen K.L., Tuck D., Parsai S., Awadallah A., Krop I.E., Winer E.P., Bossuyt V., Somlo G., Abu-Khalaf M.M., Fenton M.A., Sikov W., Harris L.N. (2016). Immune signatures following single dose trastuzumab predict pathologic response to PreoperativeTrastuzumab and chemotherapy in HER2-positive early breast cancer. Clin. Cancer Res..

[15] Herbst R.S., Baas P., Kim D.W., Felip E., Perez-Gracia J.L., Han J.Y., Molina J., Kim J.H., Arvis C.D., Ahn M.J., Majem M., Fidler M.J., de Castro G., Garrido M., Lubiniecki G.M. (2016). Pembrolizumab versus docetaxel for previously treated, PD-L1-positive, advanced non-small-cell lung cancer (KEYNOTE-010): A randomised controlled trial. Lancet.

[16] Schachter J., Ribas A., Long G.V., Arance A., Grob J.J., Mortier L., Daud A., Carlino M.S., McNeil C., Lotem M., Larkin J., Lorigan P., Neyns B., Blank C., Petrella T.M. (2017). Pembrolizumab versus ipilimumab for advanced melanoma: Final overall survival results of a multicentre, randomised, open-label phase 3 study (KEYNOTE-006). Lancet.

[17] Bertucci F., Goncalves A. (2017). Immunotherapy in breast cancer: The emerging role of PD-1 and PD-L1. Curr. Oncol. Rep..

[18] Pitt J.M., Vetizou M., Daillere R., Roberti M.P., Yamazaki T., Routy B., Lepage P., Boneca I.G., Chamaillard M., Kroemer G., Zitvogel L. (2016). Resistance mechanisms to immune-checkpoint blockade in cancer: Tumor-intrinsic and -extrinsic factors. Immunity.

[19] Nowicki T.S., Hu-Lieskovan S., Ribas A. (2018). Mechanisms of resistance to PD-1 and PD-L1 blockade. Cancer J..

[20] Loi S., Giobbie-Hurder A., Gombos A., Bachelot T., Hui R., Curigliano G., Campone M., Biganzoli L., Bonnefoi H., Jerusalem G., Bartsch R., Rabaglio-Poretti M., Kammler R., Maibach R., Smyth M.J. (2019). Pembrolizumab plus trastuzumab in trastuzumab-resistant, advanced, HER2-positive breast cancer (PANACEA): A single-arm, multicentre, phase 1b-2 trial. Lancet Oncol..

[21] Muller P., Kreuzaler M., Khan T., Thommen D.S., Martin K., Glatz K., Savic S., Harbeck N., Nitz U., Gluz O., von Bergwelt-Baildon M., Kreipe H., Reddy S., Christgen M., Zippelius A. (2015). Trastuzumab emtansine (T-DM1) renders HER2+ breast cancer highly susceptible to CTLA-4/PD-1 blockade. Sci. Transl. Med..

[22] Janjigian Y.Y., Maron S.B., Chatila W.K., Millang B., Chavan S.S., Alterman C., Chou J.F., Segal M.F., Simmons M.Z., Momtaz P., Shcherba M., Ku G.Y., Zervoudakis A., Won E.S., Kelsen D.P. (2020). First-line pembrolizumab and trastuzumab in HER2-positive oesophageal, gastric, or gastro-oesophageal junction cancer: An open-label, single-arm, phase 2 trial. Lancet Oncol..

[23] Schmid A.S., Neri D. (2019). Advances in antibody engineering for rheumatic diseases. Nat. Rev. Rheumatol..

[24] Labrijn A.F., Parren P.W. (2016). Hitting Ebola, to the power of two. Science.

[25] Wec A.Z., Nyakatura E.K., Herbert A.S., Howell K.A., Holtsberg F.W., Bakken R.R., Mittler E., Christin J.R., Shulenin S., Jangra R.K., Bharrhan S., Kuehne A.I., Bornholdt Z.A., Flyak A.I., Saphire E.O. (2016). A “Trojan horse” bispecific-antibody strategy for broad protection against ebolaviruses. Science.

[26] Labrijn A.F., Janmaat M.L., Reichert J.M., Parren P. (2019). Bispecific antibodies: A mechanistic review of the pipeline. Nat. Rev. Drug Discov..

[27] Coloma M.J., Morrison S.L. (1997). Design and production of novel tetravalent bispecific antibodies. Nat. Biotechnol..

[28] Suurs F.V., Lub-de Hooge M.N., de Vries E.G.E., de Groot D.J.A. (2019). A review of bispecific antibodies and antibody constructs in oncology and clinical challenges. Pharmacol. Ther..

[29] Brinkmann U., Kontermann R.E. (2017). The making of bispecific antibodies. MAbs.

[30] Cao M., Wang C., Chung W.K., Motabar D., Wang J., Christian E., Lin S., Hunter A., Wang X., Liu D. (2018). Characterization and analysis of scFV-IgG bispecific antibody size variants. MAbs.

[31] Schanzer J., Jekle A., Nezu J., Lochner A., Croasdale R., Dioszegi M., Zhang J., Hoffmann E., Dormeyer W., Stracke J., Schafer W., Ji C., Heilek G., Cammack N., Brandt M. (2011). Development of tetravalent, bispecific CCR5 antibodies with antiviral activity against CCR5 monoclonal antibody-resistant HIV-1 strains. Antimicrob. Agents Chemother..

[32] Delagrave S., Catalan J., Sweet C., Drabik G., Henry A., Rees A., Monath T.P., Guirakhoo F. (1999). Effects of humanization by variable domain resurfacing on the antiviral activity of a single-chain antibody against respiratory syncytial virus. Protein Eng..

[33] Gu C.L., Zhu H.X., Deng L., Meng X.Q., Li K., Xu W., Zhao L., Liu Y.Q., Zhu Z.P., Huang H.M. (2021). Bispecific antibody simultaneously targeting PD1 and HER2 inhibits tumor growth via direct tumor cell killing in combination with PD1/PDL1 blockade and HER2 inhibition. Acta Pharmacol. Sin..

[34] Ferris R.L., Jaffee E.M., Ferrone S. (2010). Tumor antigen-targeted, monoclonal antibody-based immunotherapy: Clinical response, cellular immunity, and immunoescape. J. Clin. Oncol..

[35] Yarden Y., Sliwkowski M.X. (2001). Untangling the ErbB signalling network. Nat. Rev. Mol. Cell Biol..

[36] Oh D.Y., Bang Y.J. (2020). HER2-targeted therapies - a role beyond breast cancer. Nat. Rev. Clin. Oncol..

[37] Won E., Janjigian Y.J., Ilson D.H. (2014). HER2 directed therapy for gastric/esophageal cancers. Curr. Treat. Options Oncol..

[38] Seidman A.D., Berry D., Cirrincione C., Harris L., Muss H., Marcom P.K., Gipson G., Burstein H., Lake D., Shapiro C.L., Ungaro P., Norton L., Winer E., Hudis C. (2008). Randomized phase III trial of weekly compared with every-3-weeks paclitaxel for metastatic breast cancer, with trastuzumab for all HER-2 overexpressors and random assignment to trastuzumab or not in HER-2 nonoverexpressors: Final results of cancer and leukemia group B protocol 9840. J. Clin. Oncol..

[39] Verma S., Miles D., Gianni L., Krop I.E., Welslau M., Baselga J., Pegram M., Oh D.Y., Dieras V., Guardino E., Fang L., Lu M.W., Olsen S., Blackwell K., Group E.S. (2012). Trastuzumab emtansine for HER2-positive advanced breast cancer. N. Engl. J. Med..

[40] Keam S.J. (2020). Trastuzumab deruxtecan: First approval. Drugs.

[41] Chen L., Han X. (2015). Anti-PD-1/PD-L1 therapy of human cancer: Past, present, and future. J. Clin. Invest..

[42] Ji D., Zhang J., Shen W., Du Y., Xu J., Yang J., Luo X., Kong P., Yang F., Hu X.-C. (2020). Preliminary safety, efficacy and pharmacokinetics (PK) results of KN026, a HER2 bispecific antibody in patients (pts) with HER2-positive metastatic breast cancer. J. Clin. Oncol..

[43] Lopez-Albaitero A., Xu H., Guo H., Wang L., Wu Z., Tran H., Chandarlapaty S., Scaltriti M., Janjigian Y., de Stanchina E., Cheung N.K. (2017). Overcoming resistance to HER2-targeted therapy with a novel HER2/CD3 bispecific antibody. Oncoimmunology.

[44] Junttila T.T., Li J., Johnston J., Hristopoulos M., Clark R., Ellerman D., Wang B.E., Li Y., Mathieu M., Li G., Young J., Luis E., Lewis Phillips G., Stefanich E., Spiess C. (2014). Antitumor efficacy of a bispecific antibody that targets HER2 and activates T cells. Cancer Res..

[45] Suh K.J., Sung J.H., Kim J.W., Han S.H., Lee H.S., Min A., Kang M.H., Kim J.E., Kim J.W., Kim S.H., Lee J.O., Kim Y.J., Lee K.W., Kim J.H., Bang S.M. (2017). EGFR or HER2 inhibition modulates the tumor microenvironment by suppression of PD-L1 and cytokines release. Oncotarget.

[46] Collins D.M., O'Donovan N., McGowan P.M., O'Sullivan F., Duffy M.J., Crown J. (2012). Trastuzumab induces antibody-dependent cell-mediated cytotoxicity (ADCC) in HER-2-non-amplified breast cancer cell lines. Ann. Oncol..

