# Supplementary material for: A bispecific antibody targeting HER2 and PD-L1 inhibits tumor growth with superior efficacy
Source: J Biol Chem. 2021 Nov 16;297(6):101420. doi: 10.1016/j.jbc.2021.101420 (PMC8671946; doi:10.1016/j.jbc.2021.101420)
Supplement: Supplemental Figure S1, Tables S1 and S2 [file mmc1.docx]

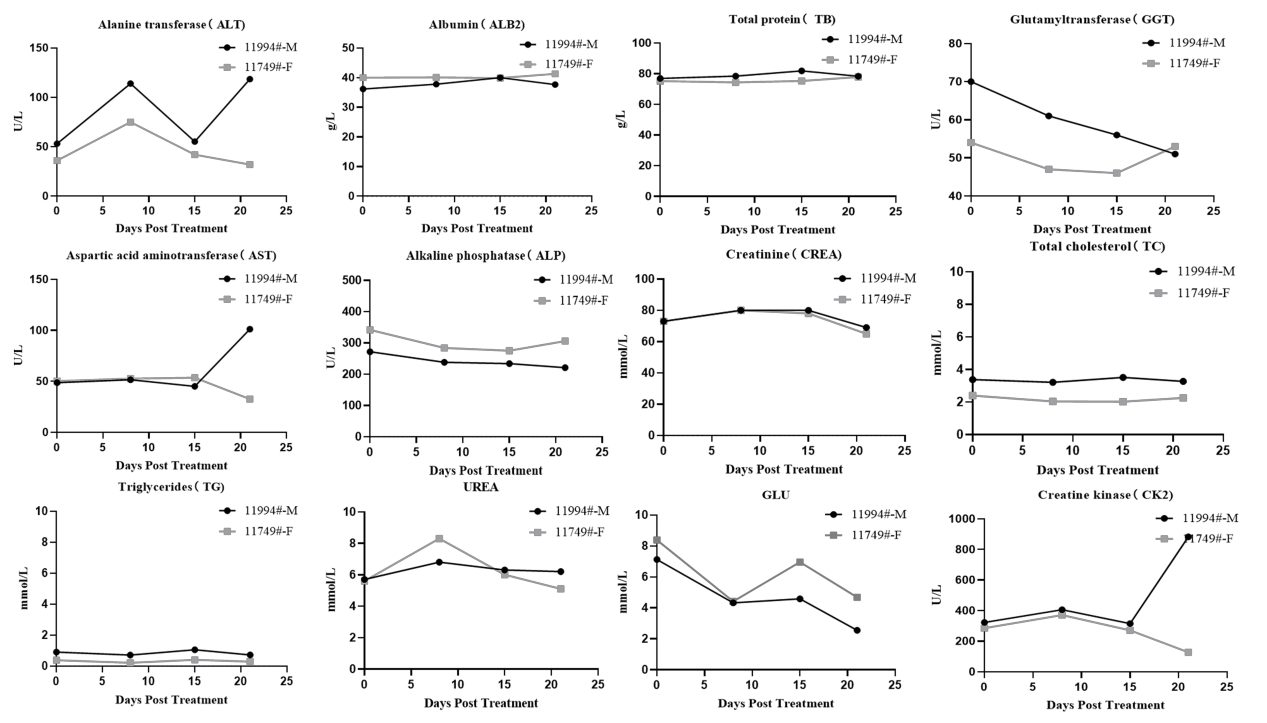


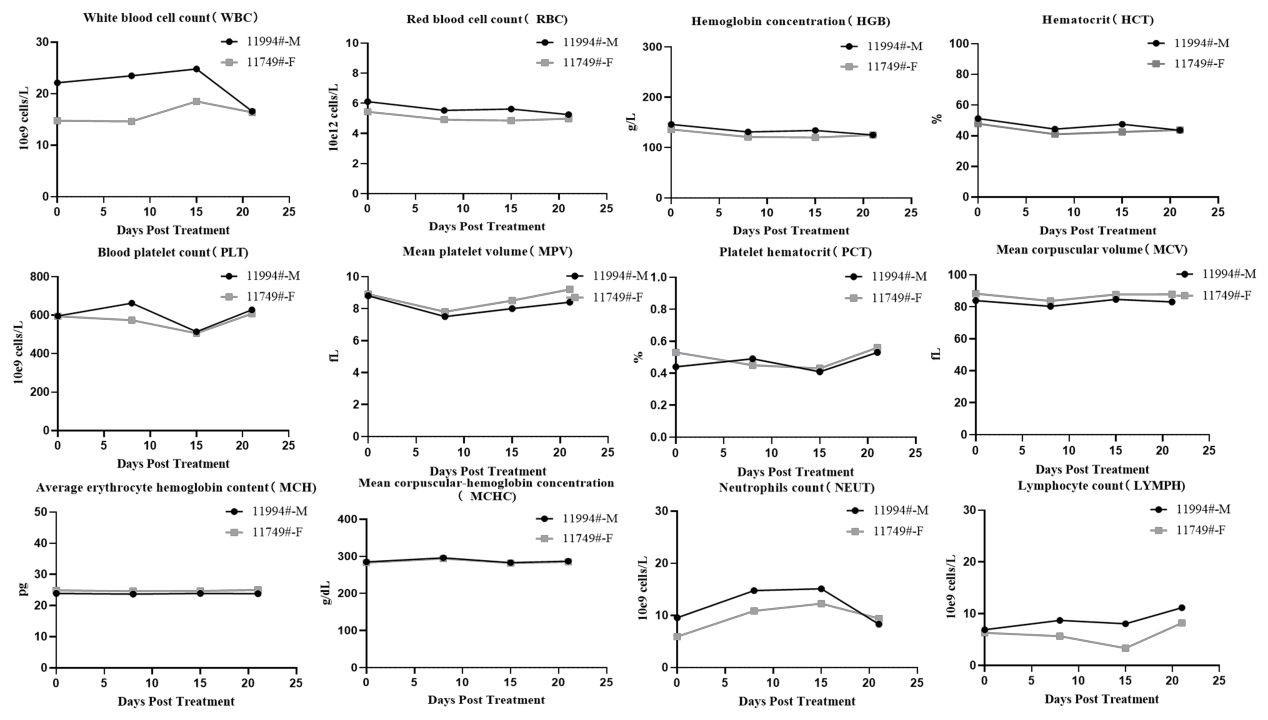


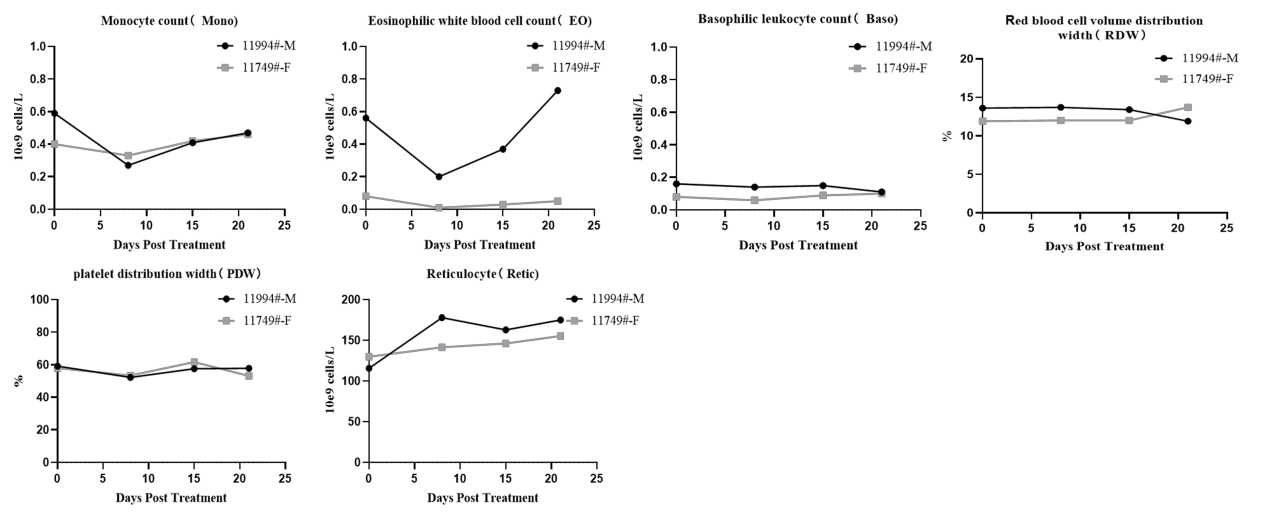


**Supplement Figure 1.**  Changes of blood cells and blood biochemical indicators after a single i.v. administration of 10 mg/kg BsAb to cynomolgus monkeys. n=2 monkeys per time point. Detected with Cobasb 221 system (Roche) and Automated Hematology Analyzer （SYSMEX).

**Supplement Table 1.** The equilibrium dissociation constant (KD) value

| **Antigen** | **Binding affinity KD (M)** | | |
| --- | --- | --- | --- |
|  | **HER2/PD-L1** | **trastuzumab** | **Avenlumab** |
| **hu-PDL1** | ＜1×10-12 | / | 1.51×10-9 |
| **hu-HER2** | 6.36×10-9 | 7.86×10-9 | / |

**Supplement Table 2.** Antitumor Activity of Test Articles in HCC1954 Xenograft Model

| **Group (n=7)** | **Tumor Volume (mm3, MeanSEM)** | | **%TGITV D41** | **RTV D41** | **Tumor weight D42** | **%TGITw D42** |
| --- | --- | --- | --- | --- | --- | --- |
|  | **D0** | **D41** |  |  |  |  |
| **1** | 149.63 ± 11.83 | 2031.13 ± 213.74 | / | 14.01 ± 1.47 | 2.074 ± 0.214 | / |
| **2** | 150.33 ± 11.22 | 949.76 ± 258.46** | 53.24 | 6.78 ± 1.98* | 0.909 ± 0.289** | 56.17 |
| **3** | 150.04 ± 11.06 | 752.59 ± 213.61** | 62.95 | 5.62 ± 1.91** | 0.756 ± 0.28** | 63.55 |
| **4** | 149.8 ± 11.15 | 667 ± 139.08*** | 67.16 | 4.93 ± 0.93*** | 0.594 ± 0.177*** | 71.36 |

*: P<0.05 compared with vehicle group; **: P<0.01 compared with vehicle group; ***: P<0.001 compared with vehicle group. Tumor Volume，TV; Tumor growth inhibition rate , TGI; Relative Tumor Volume，RTV;
